# In vivo biodistribution and toxicology studies of cadmium-free indium-based quantum dot nanoparticles in a rat model

**DOI:** 10.1016/j.nano.2018.07.009

**Published:** 2018-11

**Authors:** Elnaz Yaghini, Helen Turner, Andrew Pilling, Imad Naasani, Alexander J. MacRobert

**Affiliations:** a Division of Surgery and Interventional Science, University College London, London, UK; b Nanoco Technologies Limited, Manchester, UK; c ToxPath Consultancy Limited, Stradbroke Business Centre, Eye, Suffolk, UK

**Keywords:** Cadmium-free quantum dots, Nanoparticles, Biodistribution, Toxicology

## Abstract

Quantum dot (QD) nanoparticles are highly promising contrast agents and probes for biomedical applications owing to their excellent photophysical properties. However, toxicity concerns about commonly used cadmium-based QDs hinder their translation to clinical applications. In this study we describe the in vivo biodistribution and toxicology of indium-based water soluble QDs in rats following intravenous administration. The biodistribution measured at up to 90 days showed that QDs mainly accumulated in the liver and spleen, with similar elimination kinetics to subcutaneous administration. Evidence for QD degradation in the liver was found by comparing photoluminescence measurements versus elemental analysis. No organ damage or histopathological lesions were observed for the QDs treated rats after 24 h, 1 and 4 weeks following intravenous administration at 12.5 mg/kg or 50 mg/kg. Analysis of serum biochemistry and complete blood counts found no toxicity. This work supports the strong potential of indium-based QDs for translation into the clinic.

## Graphical Abstract

Quantum dot (QD) nanoparticles are emerging as a new class of fluorescence probes for biomedical applications. However, toxicity concerns of cadmium-based QDs hinder their translation to clinical settings. In this study we systemically investigated the in vivo biodistribution and toxicology of cadmium-free indium-based QDs in a rat model. Following intravenous injection the majority of QDs were accumulated in the liver and spleen and were excreted from the body over a period of ninety days as evidenced by elemental analysis. Comprehensive in vivo toxicology studies using histological assessments, hematological and biochemical markers demonstrated the biocompatibility of indium-based QDs for biomedical applications.

**Figure:**
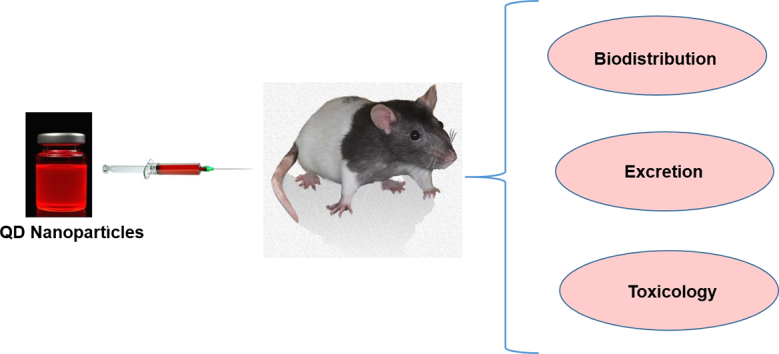

Quantum dot (QD) nanoparticles have been extensively investigated for a wide range of biomedical applications and show great promise for both imaging and therapeutic functionalities.^1^, ^2^, ^3^ However, the inherent toxicity of restricted heavy metals like cadmium severely hinders the clinical translation of such QDs owing to possible release of toxic Cd^2+^ ions, which can also induce oxidative stress via generation of reactive oxygen species (ROS).^4^, ^5^, ^6^, ^7^ This mechanism is distinct from photo-induced generation of ROS.^8^ Accumulation of nanoparticles (NPs) such as QDs in the liver and spleen following systemic administration is commonly observed and is related to the clearance of NPs from the circulation system by cells of mononuclear phagocytic system (MPS).^9^, ^10^ The in vivo fate of inorganic NPs has been recently reviewed.^11^ Elimination of NPs through the hepatobiliary system is usually slow, and solid inorganic NPs that have relatively stable cores, compared to liposomes and polymeric particles, have been shown to become sequestered in the mononuclear phagocyte system (MPS) for extended periods.^11^

Accumulation of QDs in liver, their degradation and breakdown over weeks can lead to hepatic toxicity and several in vivo studies have reported that administration of cadmium-based QDs induced morphological and functional impairments in the liver.^12^, ^13^, ^14^, ^15^ For instance, a recent study by Liu et al demonstrated that intravenous administration of CdSe/ZnS QDs in mice caused liver inflammation and dysfunction and the induced hepatotoxicity was related to the formation of ROS and the presence of cadmium.^13^ Increased levels of liver enzymes following in vivo administration of Cadmium-based QDs have been reported by several studies, suggesting impaired liver function/or hepatocellular injury.^14^, ^15^, ^16^ Although Hauck et al found that CdSe/ZnS QDs did not induce appreciable toxicity in Sprague-Dawley rats over the course of their study,^17^ Tiwar et al observed significant toxicity of CdSe/ZnS QDs in Wistar rats,^16^ including higher liver enzymes and liver inflammation. In a study by Roberts et al, lung injury and inflammation were reported following administration of CdSe/ZnS QDs in rats.^18^ Likewise Ho et al noted persistent inflammation and granuloma formation in the mouse lung following administration of cadmium-based QDs.^19^ In a study by Tang et al higher levels of creatinine (Cr) were observed after mice were injected with cadmium-based QDs which were attributed to kidney injury.^15^ In addition, hyperplasia and swelling of glomerular capillary endothelium were observed in the H & E stained sections of the kidney.

In order to address toxicity concerns regarding the cadmium content, considerable effort has been expended in fabricating cadmium-free QDs, as recently reviewed.^20^ A variety of cadmium-free QDs have been developed with cores composed of materials such as silver sulfide (Ag_2_S), silver selenide (Ag_2_Se), silver indium sulfide (AgInS_2_) and copper indium sulfide (CuInS_2_).^20^ Several types of indium phosphide-based QDs have also been developed either with InP cores or alloys with other core constituents such as InPZnS.^21^, ^22^, ^23^, ^24^ Cadmium-free QDs composed of group III-V semiconductor nanocrystals (particularly InP) are structurally more robust and stable owing to the presence of covalent bonds in their matrix.^25^, ^26^ The photoluminescence (PL) intensity and the quantum yield (QY) of InP QDs have been generally lower than cadmium-based QDs. However, several studies have shown that coating the InP core with a shell of a wider band gap semiconductor increases the QY. Indium-based QDs are therefore emerging as a promising substitute for cadmium-based QDs for biological studies.^22^ Nevertheless, there are currently only limited data on the short-term to long-term in vivo biodistribution and toxicology of indium-based QDs, which need to be addressed prior to clinical evaluation.

Recently, we reported a new type of indium-based QDs, bio CFQD® nanoparticles, and demonstrated their potential for in vivo sentinel lymph node (SLN) imaging^27^ following subcutaneous injection. In the present work we carried out systematic testing in a rat model to investigate the short-term and long-term pharmacokinetics, biodistribution and blood clearance of QDs following their intravenous injection. To elucidate the in vivo toxicity of the nanoparticles a comprehensive analysis was performed at two different doses, in order to discern any escalation in response.

## Methods

### Synthesis and characterization of bio CFQD® nanoparticles

The synthesis of the indium-based QDs (bio CFQD® nanoparticles) was performed at the laboratories of Nanoco Technologies, Limited, Manchester, UK^27^ using proprietary synthesis procedures based on the molecular seeding process.^28^ Briefly, a ZnS molecular cluster [Et_3_NH_4_][Zn_10_S_4_(SPh)_l6_] was heated in the presence of indium myristate (In (MA)_3_) and Tris(trimethylsilyl)phosphine ((TMS)_3_P) in a medium of di**-**n**-**butylsebacate ester. Then the resulting indium-based alloyed cores were washed with HF and heated in a solution of zinc acetate and Bis(trimethylsilylmethyl) sulfide ((TMS)_2_S) in butylsebacate ester to form a coating layer of ZnS. Finally, the organic surface coating and functionalization were conducted using the hexamethoxymethylmelamine (HMMM) method,^29^ as described in our earlier study.^27^ For purification, crude aqueous nanoparticles from the surface coating process were purified by multiple cycles of ultrafiltration using 30 kDa centrifugation filters. Aqueous stock solutions of the nanoparticles were stored at 4 °C. The resulting water soluble bio CFQD® nanoparticles had an average hydrodynamic diameter of 12.2 nm as determined by dynamic light scattering (Malvern Zeta Sizer), a surface grafted with polyethylene glycol (MW 2000), and a negative charge resulting from surface COOH groups. The diameter of the central inorganic component of the QD was c. 4 nm as determined from transmission electron microscopy (TEM).^27^

**Figure 1:**
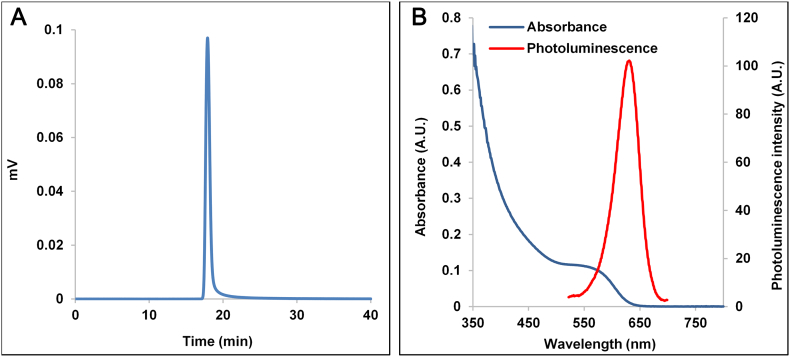
**(A)** Size exclusion chromatography profiles of bio CFQD® nanoparticles. **(B)** Absorbance and fluorescence spectra of bio CFQD® nanoparticles in aqueous solution.

Mono-dispersity of the particles and the absence of aggregates and particulate impurities were confirmed using size exclusion chromatography (SEC, Waters) (Figure 1). The used column was an Agilent PEO/PEG type (PL aquagel-OH MIXED-H) with deionized water as a mobile phase at 1 mL/min flow rate. A 10 μL sample containing 10 μg of QD material was injected and the detection used dual UV absorbance at 278 nm and photoluminescence at 450 nm excitation and 625 nm emission. The elution profiles of both absorbance and photoluminescence traces overlapped as a single peak, indicating that the final QD material is free of fluorescent and non-fluorescent aggregates, and confirm the high purity. The spectral properties of the final particles are shown in Figure 1, *B*. The photoluminescence spectrum was recorded using a Hamamatsu Absolute Photoluminescence Quantum Yield spectrometer (C9920-2, Hamamatsu Photonics K.K) with excitation at 450 nm. The QDs exhibit good fluorescence quantum yields (0.35-0.45) in aqueous buffers (pH 5-8).

### Animal experiments

Female Lister Hooded rats were purchased from Harlan UK Ltd. All procedures were carried out with Home Office license approval.

### Biodistribution and pharmacokinetic studies

Ten groups of animals (n = 5) were used: nine groups were injected intravenously via the tail vein with 500 μL of bio CFQD® nanoparticles solution in PBS at a concentration of 12.5 mg/kg, with one as the control injected with 500 μL of PBS. Rats were sacrificed at 5 min, 1 h, 4 h, and 1, 3, 10, 30, 60 and 90 days and blood and various organs were collected.

### Quantification of uptake in organs

0.1 g of each tissue in triplicate was prepared and digested by the addition of 1 mL 70% nitric acid (HNO_3_) as detailed previously^27^ Inductively coupled plasma-mass spectroscopy (ICP-MS) was utilized to quantify the amount of indium (In) in the organs. For all measurements, nitric acid blank, blank tissue samples, spiked samples with known QDs for calibration and indium standards were prepared and tested concurrently with test samples. The tissues from the control rats without QD administration were dissolved in a similar manner.

### In vivo toxicology study

Nine groups of animals (n = 5) were used: six groups were injected intravenously with 500 μL of bio CFQD® nanoparticle solution in PBS at a concentration of 12.5 mg/kg and 50 mg/kg, and three as control groups injected intravenously with 500 μL PBS. The rats were sacrificed at 24 h, 1 week and 4 weeks after the injection and blood and serum samples were collected for full biochemical and hematological analysis. Major organs including liver, spleen, kidney, brain, thymus, lung, heart, mesenteric lymph nodes were removed, fixed in 4% formalin, sectioned, and stained (Hematoxylin & Eosin). Analysis of tissues was performed by a veterinary pathologist.

### Cryosection fluorescence microscopy

Rats were administered intravenously with bio CFQD® nanoparticles at 12.5 mg/kg. Animals were sacrificed at various post-injection times and the liver was harvested for microscopic imaging of QD photoluminescence and snap frozen with an isopentane slush and then stored at −80 °C. Five consecutive 10 μm thick tissue sections from each tissue block mounted with OCT Embedding Medium (Raymond A. Lamb, UK) were cut. Photoluminescence was recorded quantitatively using an inverted epifluorescence microscope (Olympus IMT-2) equipped with a 16 bit cooled 512 × 512 pixel CCD camera (Princeton Instruments, model PIXIS 512F). Excitation was provided by a 405 nm laser diode module (Laser Components UK, Ltd), and emission detected via a 500 nm cut-on dichroic mirror and 40 nm bandpass emission filter centered at 620 nm close to the peak emission of the QDs (Omega Optical Inc. 500 AGSP and 620DF40), and a long pass filter (Schott RG570). The mean signal intensity of the microscopic field (500 × 500 μm) of each tissue section was analyzed using ImageJ software (NIH, US).

### Statistical analysis

Statistical analysis was performed with a two-sample *t* test with unknown and unequal variances, comparing each sample group to the related control group. The error bars shown are the standard deviations (SD). Results were considered significant for *P* < 0.05.

## Results

### In vivo biodistribution

**Figure 2:**
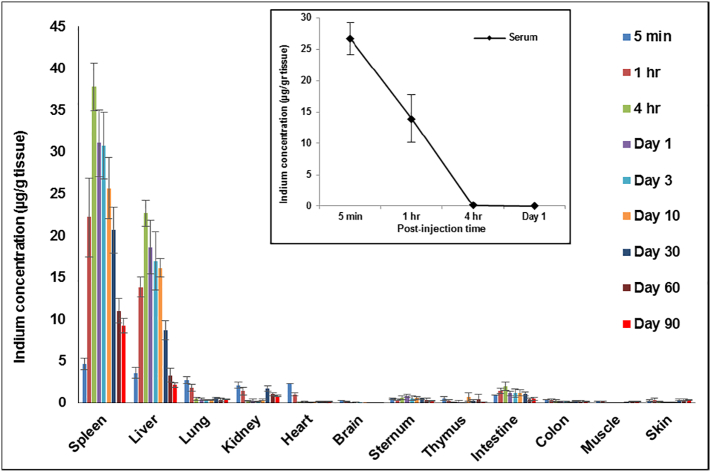
In vivo biodistribution analysis over a period of 90 days in Lister Hooded rats following administration of bio CFQD® nanoparticles. The indium concentration in the organs was determined at different time points after intravenous injection of bio CFQD® nanoparticles (12.5 mg/kg) using ICP-MS (n = 5). The inset shows the decay in circulating serum levels in the same animals.

In vivo biodistribution and toxicology studies using rats have several advantages stemming from their larger size and physiology compared to mice, and in this study we investigated the short-term and long-term in vivo biodistribution of the bio CFQD® nanoparticles in rats following intravenous administration at a dose of 12.5 mg/kg. We observed that 5 min post-injection the majority of QDs were in the circulation (26 μg In/g) and only small amounts of indium were detected in all other tissues (Figure 2). At 1 h post-injection, a two-fold decrease of indium concentration in the serum was observed and QDs were predominantly accumulated in the liver and spleen where the indium content in the liver and spleen was found to be 13 μg In/g and 22 μg In/g respectively. The concentration of indium in liver and spleen peaked at 4 h post-injection (Liver: 22 μg In/g and spleen: 37 μg In/g). The increase in indium concentration in the liver and spleen was consistent with the decrease of indium in circulation over the same time span. After 4 h, levels in the liver and spleen steadily decreased and by 90 days less than 15% of the 4 h peak level remained in the liver. Trace quantities of indium were detected in all other tissues at various post-injection times, notably the intestine. Small amounts were present in the sternum samples which included the bone marrow. In the kidney, a small amounts were detected but levels increased slightly at 30 days and longer. Levels in the brain were negligible.

**Figure 3:**
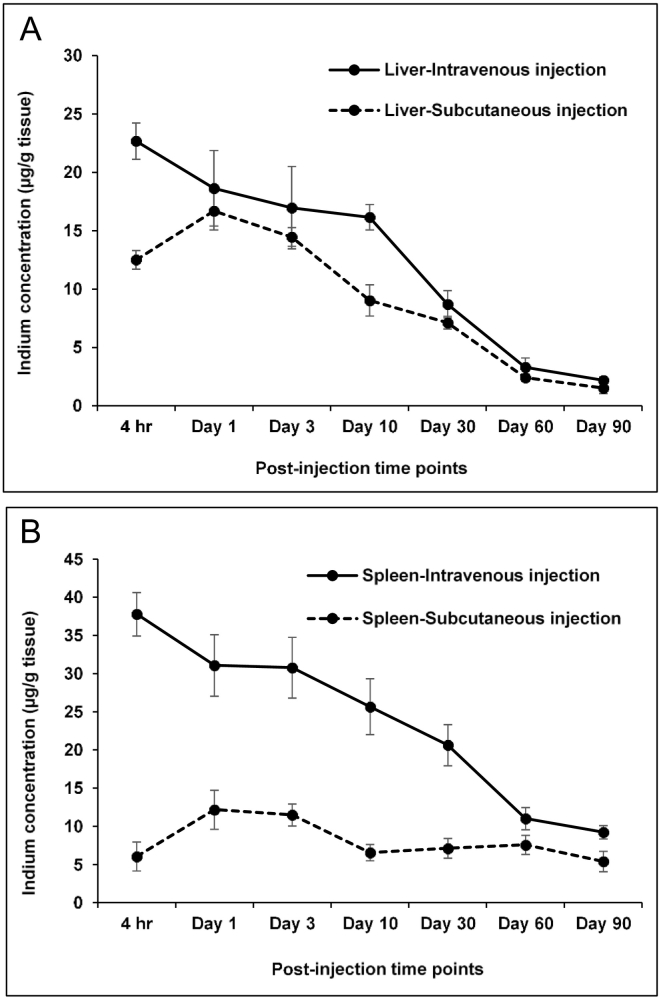
In vivo biodistribution analysis of indium levels over a period of 90 days in Lister Hooded rats following intravenous (12.5 mg/kg) and subcutaneous (7.5 mg/kg) injection of bio CFQD® nanoparticles in **(A)** liver tissues and **(B)** spleen tissues. The indium concentration in the organs was determined at different post-injection time points using ICP-MS. The data for subcutaneous injection are replotted from our previous study.^27^

We recently reported biodistribution studies following subcutaneous administration at 7.5 mg/ kg using the same nanoparticles in the same strain of rats which enables us to draw comparisons between the two routes.^27^ The highest levels accumulated in the axillary and thoracic regional lymph nodes near the injection site in the paw with lower levels in the liver and spleen and only negligible amount of indium was detected in all other tissues. The dose used is lower than used in the present study with intravenous administration (12.5 mg/kg). Figure 3 shows the data for liver and spleen from our previous study using subcutaneous administration together with the intravenous administration data. Peak levels in the liver and spleen were observed at 1 day using subcutaneous injection in contrast to the intravenous studies where accumulation was higher in the liver and spleen at 4 h

### Biodistribution of QDs in rat liver using cryosection microscopy

**Figure 4:**
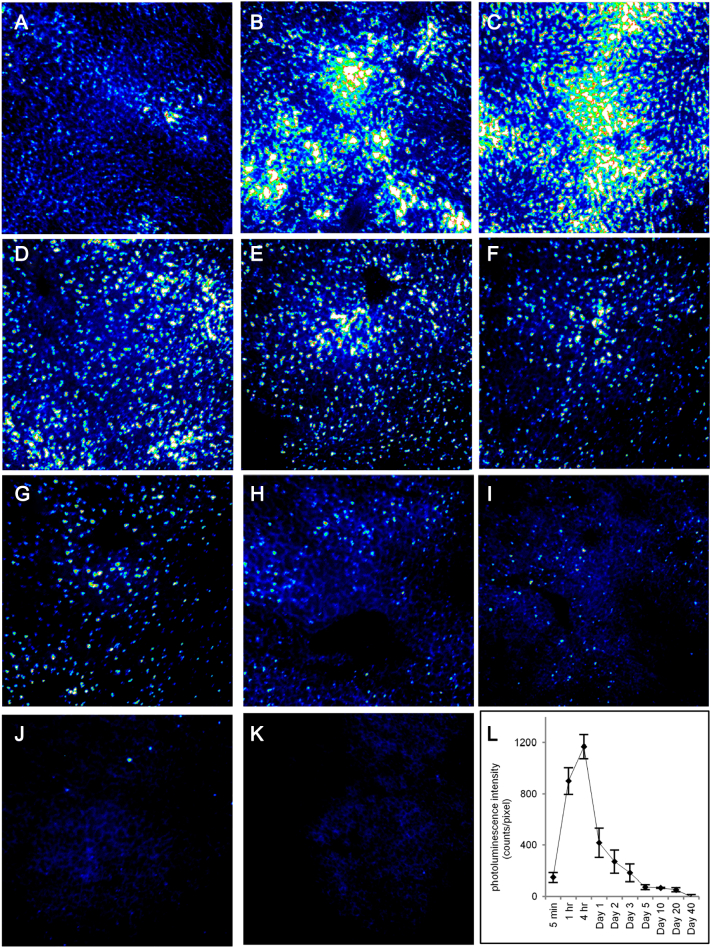
Tissue cryosection fluorescence microscopy showing distribution of photoluminescence from bio CFQD® nanoparticles in the rat liver. Blue corresponds to lower intensity and white to highest, and the same intensity scale was used throughout for direct comparison. QDs were injected at 12.5 mg/kg intravenously and images were obtained at various time intervals post-injection. **(A)** 5 min; **(B)** 1 h; **(C)** 4 h; **(D)** 24 h; **(E)** 48 h; **(F)** 72 h; **(G)** 5 days; **(H)** 10 days: **(I)** 20 days: **(J)** 40 days: **(K)** control - without QDs injection. **(L)** Time-course of integrated mean photoluminescence intensity of bio CFQD® nanoparticles in the cryosections of rat liver using fluorescence microscopy with control baseline level subtracted.

Quantitative fluorescence microscopy was used to image the photoluminescence of bio CFQD® nanoparticles in liver cryosections taken from rats injected intravenously with QDs at 12.5 mg/kg. Figure 4 displays cryosection fluorescence microscopy images of the rat as a function of time after administration. Compared to the control group, QDs present in liver sections exhibited a bright punctate photoluminescence pattern which was distributed evenly throughout the liver as early as 5 min after injection. The photoluminescence intensity appears to decrease gradually over time although the punctate pattern was maintained, though sparser, and reached baseline levels by 40 days post-injection.

To study the time-course of the QDs photoluminescence intensity quantitatively in the liver, the mean photoluminescence intensity across each image field was measured for multiple sections taken from several animals (n = 3), including correction for weak tissue autofluorescence. Since liver is relatively homogenous this technique is reproducible from sample to sample, and we have used the same technique previously to study photosensitizer dye biodistributions quantitatively.^30^, ^31^ As shown in Figure 4, *L*, the integrated photoluminescence of the QDs was maintained at shorter times following administration. However the intensity then declined noticeably post-injection reaching basal levels at 40 days.

### In vivo toxicology

In this study, we systemically investigated the toxicity of bio CFQD® nanoparticles in rats following intravenous injection at either 12.5 or 50 mg/kg (n = 5). The control rats were injected intravenously with PBS. Rats were sacrificed at 24 h, 1 week and 4 weeks after QDs injection for blood and organ collection. Throughout the study QD-administered rats did not exhibit unusual behavior or responses compared to control rats. The body weight of the control group and the two experimental groups of rats exhibited comparable increasing trends over 5 weeks post-injection (data not shown) suggesting that QDs did not interfere with the growth rate of the animals.

**Figure 5:**
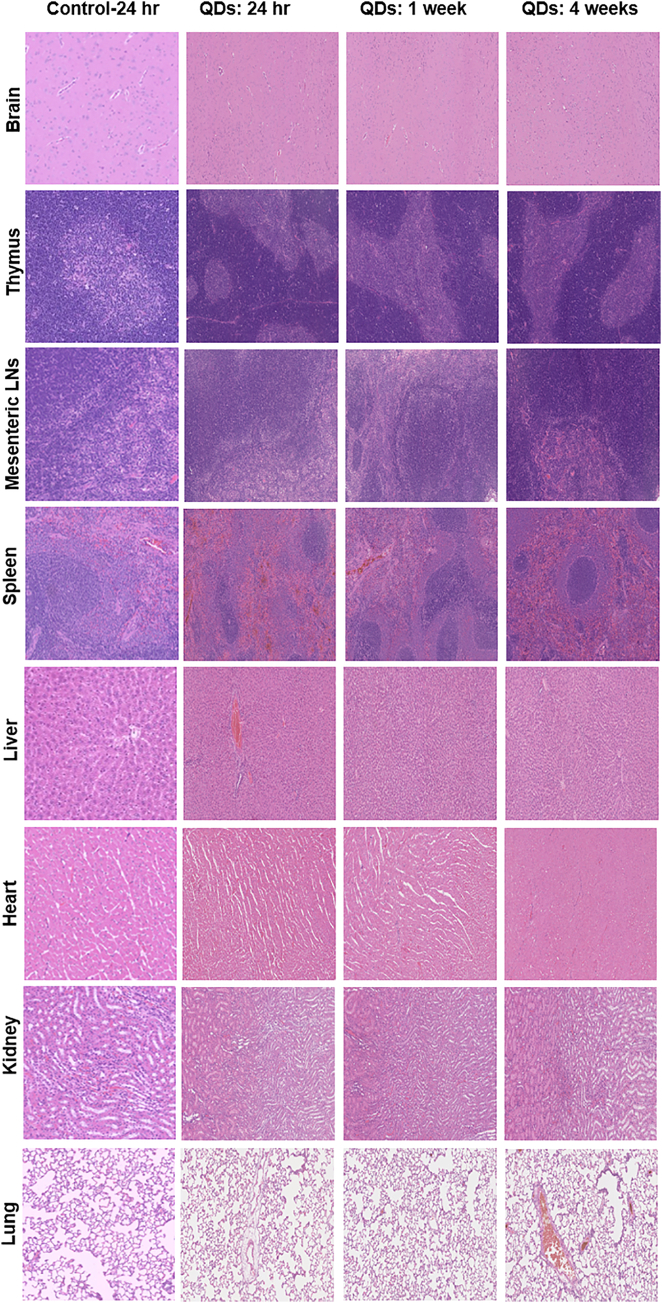
Representative organic histology, the H&E stained images of major organs including brain, thymus, mesenteric lymph nodes, spleen, liver heart, kidney and lung collected from the control untreated rats and QD injected rats following intravenous injection at concentration of 50 mg/kg at 24 h, 1 week, and 4 weeks post-injection.

Histological assessment was performed to examine tissue damage, inflammation and lesion from QDs exposure. Representative histology results are shown in Figure 5 for the higher 50 mg/kg QD dose. Accumulation of cadmium-based QDs in the liver and spleen can cause adverse effects including pathological changes in their morphology and infiltration of inflammatory cells.^12^ In our study, no sign of an inflammatory response or pathological changes was observed in liver and spleen. Elemental analysis (Figure 2) showed relatively low amounts of indium in the kidneys but levels increased slightly at 30, 60 and 90 days post-injection, which might be due to free indium excretion following QD degradation. Importantly, histological analysis of the kidney (including the glomeruli) did not indicate any histopathological changes. In the lung, QDs have been reported to cause inflammation, granuloma formation and lung dysfunction.^15^, ^18^, ^19^, ^32^, ^33^ No pulmonary histological changes were detected in our study. In all the other tissues studied including brain, thymus and mesenteric lymph nodes no histopathological abnormalities were observed.

**Figure 6:**
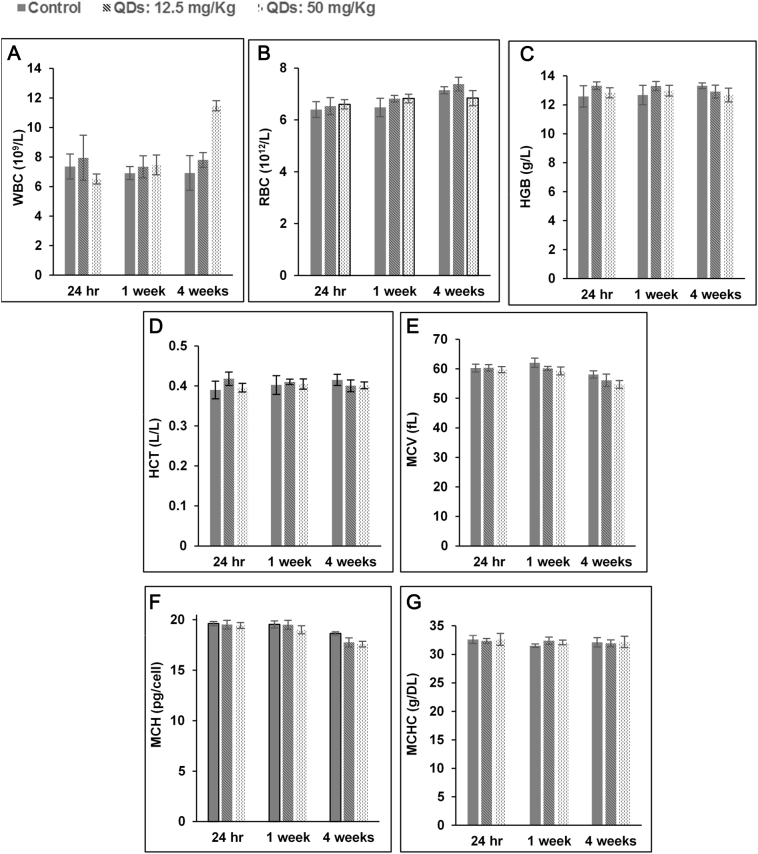
Hematology results of the bio CFQD® nanoparticles injected intravenously into the rats (n = 5). The results show mean and standard deviation of white blood cells (WBC), red blood cells (RBC), hemoglobin (HGB), hematocrit (HCT), mean corpuscular volume (MCV), mean corpuscular hemoglobin (MCH), mean corpuscular hemoglobin concentration (MCHC).

NPs can have adverse effects on hematological markers, and thus hematological parameters are an important part of assessment for the clinical applications of NPs.^34^, ^35^, ^36^ For all the hematological markers, except the WBC count, levels in the QDs injected groups remained statistically indistinguishable from the control group at all time points and injected doses (Figure 6). The WBC increased in QD-treated rats injected with 50 mg/kg at 4 weeks post-injection. However, no significant change in WBC was observed in rats administered with QDs at 12.5 mg/kg. A comparative hemolysis study was also performed where the QDs used herein were compared to commercially available CdSe/ZnS QDs (Supplementary Data, Figure S1).

**Figure 7:**
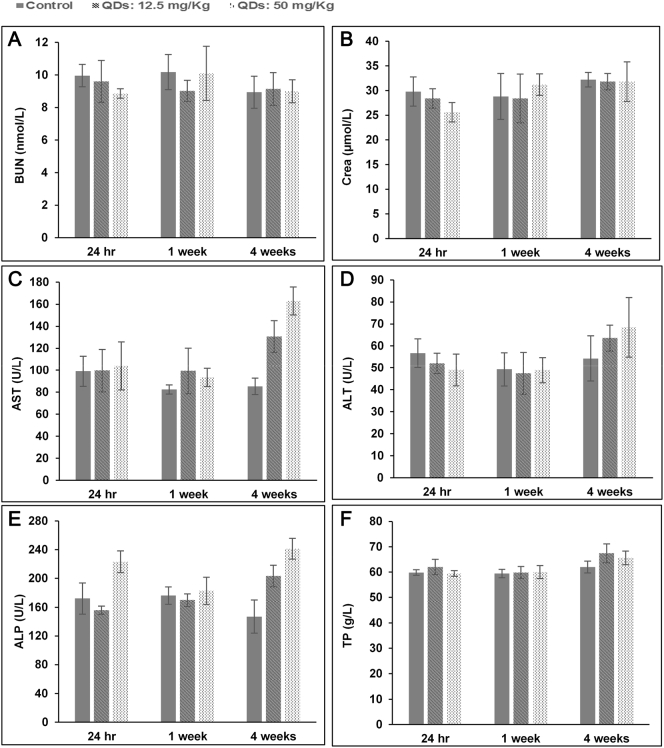
Blood biochemical results of the nanoparticles following intravenous into rats (n = 5). The results show mean and standard deviation of **(A)** blood urea nitrogen (BUN), **(B)** creatinine (Crea), **(C)** aspartate transaminase (AST), **(D)** alanine transaminase (ALT), **(E)** alkaline phosphatase (ALP), **(F)** total protein.

Clinical biochemistry tests were performed to evaluate organ function. As shown in Figure 7, the amount of total protein, albumin and total bilirubin (data not shown for total albumin and bilirubin) showed similar trends to those from the control group. Slightly higher levels of ALT, AST, and ALP compared to the control group were observed at 4 weeks post-injection time points. Nevertheless, at shorter time-points (24 h and 1 week after injection) the levels were similar to the control ones. Kidney function was assessed by measuring blood urea nitrogen (BUN) and creatinine (Cr). In our study, the levels of BUN and Cr in QD treated groups were similar to the control groups suggesting that there was no renal dysfunction.

## Discussion

To date, relatively few studies have used rats for in vivo biodistribution and toxicology assessment of QDs. However, for such studies rat models have several advantages over mice, including practical advantages of larger blood volumes and organ sizes for histological and biochemical analysis. Use of the larger animal requires the preparation of larger amounts of QDs and in turn reproducible synthesis in bulk quantities. As shown in Figure 2, there was relatively high accumulation of indium in the liver and spleen measured using elemental analysis. After peaking at 4 h, indium levels in the liver and spleen steadily decreased so that by 90 days post-injection only small amounts remained. The progressive clearance of indium is due to excretion of the QDs presumably via the hepatobiliary route as well as any excretion of small degradation products of QDs containing indium by renal clearance. Trace quantities of indium were detected in the intestine and colon, showing that some QDs or degradation products containing indium were cleared through the hepatobiliary route. In a study by Peracchi et al PEGylation of the NPs decreased the hepatic accumulation of the NPs whereas increased spleen uptake was observed and the authors suggested that it is more likely that a fraction of PEGylated NPs that avoid uptake in the liver are delivered to the spleen.^37^, ^38^ In our study, the surface of the bio CFQD® nanoparticles were grafted with PEG which may explain the higher splenic uptake of these QDs. Similar trends have been observed following intravenous injection of PEGylated InP/ZnS QDs in mice wherein spleen exhibited higher uptake of QDs than the liver.^39^

The size of the QDs used in our study with a 12.2 nm hydrodynamic diameter^27^ is above the threshold for direct renal clearance,^40^ and only small quantities of indium were detected in the kidney initially. Subsequently, a small but progressive increase in the amount of indium in the kidney was observed at 30 days, 60 days and 90 days post-injection (1.6, 1 and 0.8 μg In/g, respectively). This suggests some QDs underwent intracellular degradation and also released degraded precursors containing indium for renal excretion. In all other tissues including heart, thymus and skin, trace quantities of indium were detected with no significant trend. The circulation half-life (τ_1/2_) of QDs is ~ 1 h based on the indium concentration, shown in the inset of Figure 2. We attribute the fast clearance from the blood to liver and spleen uptake. Biocompatible polymers such as polyethylene glycol (PEG) can only inhibit but not prevent MPS uptake of NPs, and rapid clearance of PEGylated QDs from circulation system has also been reported with other PEGylated QDs. For instance, in the studies of Tang et al, PEGylated Ag_2_Se QDs with a hydrodynamic diameter of 29 nm were cleared from mice blood circulation quickly following intravenous injection.^41^ The blood circulation half-life of these QDs was 0.4 h based on chemical extraction (Ag ion concentration). In the study reported by Lin et al, PEGylated InP/ZnS QDs injected intravenously in mice were also cleared from circulation rapidly with the majority cleared within 4 h.^39^ However the hydrodynamic diameter was much larger at 58 nm compared to the bio CFQD® nanoparticles used herein, c. 12.2 nm.

We recently carried out biodistribution studies following subcutaneous administration of the same type of QD at a dose of 7.5 mg/kg injected into the rat paw using the same nanoparticles in the same strain of rats which enables comparison between the two routes of administration.^27^ As shown in Figure 3, peak levels in both liver and spleen using subcutaneous injection were reached at 1 day whereas accumulation was higher in the liver and spleen at 4 h than 1 day with intravenous administration. The slower rate of accumulation using the subcutaneous route reflects the slower migration of the QDs to these organs from the injection site in the paw. Despite the lower dose, subcutaneous injection elicited comparable uptake in the liver but significantly lower uptake in the spleen by a factor of seven whereas the doses differ by less than a factor of two. Comparing the indium contents in the liver and spleen over the period of 90 days, it appears that the rates of elimination from day 1 onwards up to day 90 in the liver and spleen following subcutaneous injection were similar to those of intravenous injection. For example, for liver at 30 days compared to 24 h, there was a 53% decline in mean levels for the intravenous route versus a 57% decline for the subcutaneous route. At 90 days the levels had declined by 88% and 91% respectively. For spleen the corresponding figures are 34% and 42% at 30 days, and 70% versus 56% at 90 days, showing slower but similar elimination rates. This indicates that the excretion rates were not strongly correlated with the injection method.

Tsoi et al have shown that in the rat liver intravenously administered PEGylated QDs mainly accumulated in the Kupffer cells, hepatic B cells and liver sinusoidal endothelial cells and suggested that Kupffer cells were primarily responsible for the nanoparticle clearance from the liver.^11^ As shown in Figure 4, a punctate photoluminescence pattern was observed in microscopic images of liver cryosections, which was maintained at later times but with decreasing intensity. It is likely that this punctate pattern was primarily due to phagocytic uptake by Kupffer cells and possibly liver sinusoidal endothelial cells (LSEC).^35^, ^42^, ^43^ In a recent study, Liang et al reported that intravenously injected mercaptosuccinic acid (MSA)-capped CdTe/CdS QDs are retained in liver sinusoids and selectively are taken up by sinusoidal cells (Kupffer cells and liver sinusoidal endothelial cells), but not hepatocytes within 3 h.^44^ In our study, the reduced photoluminescence intensity levels in liver cryosections at longer post-injection time points must be partially related to excretion of QDs from the body, but different trends were noted between elemental analysis measurements and photoluminescence intensities. For instance, the elemental analysis (Figure 2) still showed relatively high indium accumulation in the liver at 10 days post-injection, whereas cryosection microscopy demonstrated weak photoluminescence signals from the QDs at 10 days post-injection time-points (Figure 4, *H*). It is likely that some of the reduction of photoluminescence is due to intracellular degradation by reactive oxidizing species in compartments of phagocytic cells present in liver sinusoids, particularly Kupffer cells.^11^ Mancini et al suggested that oxidative etching of the quantum dot shell may be induced by hypochlorous acid for example which is present at relatively high concentrations in phagocytic cells.^45^ We are assuming as in previous studies that cryosection preparation process does not impair the PL efficiency as a function of the QD residence time in the tissue.^46^ We can draw further comparison with our previous study on the biodistribution of the bio CFQD® nanoparticles following subcutaneous injection in rats, where we observed bright and relatively stable photoluminescence in regional lymph nodes up to 10 days.^27^ The photoluminescence intensity profile with time matched the elemental analysis of indium levels in contrast to the liver data in the present study. It is possible that more rapid degradation of the QDs may take place in the liver due to the different localization and properties of the phenotype of the phagocytes present in the liver such as Kupffer cells.^11^

In our previous studies of these QDs using subcutaneous injection in rats, we did not observe any adverse effects to the animals but did not specifically examine in vivo toxicology.^27^ In this study we systematically investigated the toxicity of indium-based QDs after intravenous injection to rats at two doses, 12.5 or 50 mg mg/kg. In an in vivo study on regional lymph node uptake in mice, Pons et al carried out histological analysis on sections of lymph nodes after subcutaneous injection of CdTeSe/ZnS QDs in mice at up to 7 days post-injection and observed an inflammatory response in axillary lymph nodes at a ten-fold lower dose compared to administration of cadmium-free CuInS_2_/ZnS QDs.^47^ A comparative study of water soluble CdSe/ZnS and InP/ZnS QDs using the *Drosophila* fruit fly model reported significantly higher toxic effects with the cadmium-based QDs, owing primarily to the release of the toxic Cd^2+^ ions. Although comparable amounts of indium ions were also released from the indium-based QDs, their lower intrinsic toxicity appeared to negate any toxic effects.^22^ Comparison of in vitro hemolysis properties using blood samples from the rats (Supplementary Data, Figure S1) showed that the indium-based QDs studied herein elicited negligible (<10%) hemolysis at the highest concentration studied compared to commercially available CdSe/ZnS QDs for which c. 90% hemolysis was observed. This is consistent with lack of toxicity evident using the standard MTT assay on the same QDs incubated with MCF-7 human breast carcinoma cells.^27^

As summarized earlier, there are several reports of toxicity observed in the liver, lungs and kidney following intravenous administration of cadmium-based QDs.^12^, ^13^, ^14^, ^15^, ^16^, ^18^ There has also been a report of slight edema and necrosis in liver in mice following administration using a dose of 8 μmol/kg PEGylated Ag_2_Se QDs with a hydrodynamic diameter of 29.^41^ These data contrast with the present study using indium-based QDs where histopathological assessment of liver and spleen tissues revealed no sign of an inflammatory response or pathological changes in liver and spleen despite the relatively high uptake in these organs (Figure 5). In our study using the indium-based QDs no inflammation was noted in the lungs. Histological analysis of the kidney did not show any histopathological changes. Likewise in the other tissues, including brain, thymus and mesenteric lymph nodes, no histopathological abnormalities were observed. Kidney function was assessed by measuring blood urea nitrogen (BUN) and creatinine (Cr). In this study, the levels of BUN and Cr in QD treated groups were comparable to the control groups suggesting that there was no renal dysfunction (Figure 6).

Our studies showed that a slight increase in plasma levels of liver enzymes (ALT, AST and ALP), in association with increased WBC (at 50 mg/kg), at the 4-week time-point which is consistent with a low-grade, sub-lethal disturbance of hepatocytes (Figure 6, Figure 7). The minor nature of this injury is confirmed by the absence of microscopic changes and the presence of normal circulating total protein/albumin levels, which indicate no alteration in liver function. These enzymatic changes are probably due to the degradation and breakdown of QDs following their accumulation in the liver. The small increase in WBC is possibly in response to events in the liver at this time-point although no microscopic changes were observed. Increases in the number of WBC have been reported in other studies following intravenous injection of QDs which have been attributed to the inflammatory response.^15^ All other hematological markers in the QDs injected groups remained statistically indistinguishable from the control group at all time points and injected doses (Figure 6).

In summary, most toxicological and biodistribution studies of quantum dots to date have been conducted in mice. This work presents a detailed study of the toxicology and biodistribution of indium-based quantum dots in rats following intravenous tail vein injection. Following administration, the QDs mainly accumulated in the liver and spleen and were excreted from the body gradually as observed over a period of ninety days using elemental analysis, supplemented by photoluminescence imaging in the liver which indicated that QD degradation occurred in liver. Comparison with our previous studies using the same type of QD in the same strain of rat using subcutaneous injection showed that the elimination rates from liver and spleen were similar, although much lower accumulation in the liver and spleen occurred with sub-cutaneous administration. Systematic in vivo toxicology studies including histological, biochemical and hematological parameters demonstrated the biocompatibility of bio CFQD® nanoparticles for biomedical applications.

## Appendix A Supplementary data

Supplementary material Image 1
