# Supplementary material for: In vivo biodistribution and toxicology studies of cadmium-free indium-based quantum dot nanoparticles in a rat model
Source: Nanomedicine. 2018 Nov;14(8):2644–55. doi: 10.1016/j.nano.2018.07.009 (PMC6198065; doi:10.1016/j.nano.2018.07.009)
Supplement: Supplementary file 1 — Supplementary material [file mmc1.docx]

**In vivo biodistribution and toxicology studies of cadmium-free indium-based quantum dot nanoparticles in a rat model**

Elnaz Yaghini, MD, PhD ^a,*^, Helen Turner, BSc^b^, Andrew Pilling, PhD^c^,

Imad Naasani, PhD^b^, Alexander MacRobert, PhD^a^

^a^Division of Surgery and Interventional Science, University College London, London, UK

^b^Nanoco Technologies Limited, 46 Grafton Street, Manchester, UK

^c^ToxPath Consultancy Limited, Stradbroke Business Centre, Eye, Suffolk, UK

^*^Corresponding author: Dr Elnaz Yaghini, Division of Surgery and Interventional Science, University College London, London, UK

Email address: [elnaz.yaghini@ucl.ac.uk](mailto:elnaz.yaghini@ucl.ac.uk)

**In vitro haemolysis study**

The haemolysis assay was performed to investigate in vitro haemolysis of bio CFQD^®^ nanoparticles. Fresh blood samples were collected from female Lister Hooded rats. QDs solutions at various concentrations were prepared (12.5 - 200 nM) and 1 mL aliquot of diluted blood (0.2 mL in 10 mL 0.9% sodium chloride) was added to each Eppendorf tubes. The samples were then incubated at 37 °C. To investigate the effect of time on red blood cell (RBC) lysis in the presence of QDs, blood was incubated with QDs for up to 5 hr (1 hr, 2 hr, 3 hr, 4 hr and 5 hr). After desired incubation time, the samples were centrifuged for 10 min at 1000 g. Supernatant was collected and 100 µL from each samples was added into the each well of a 96 well-plate. The absorbance of the supernatant (oxyhaemoglobin) was measured at 565 nm using a plate reader. Red blood cells suspended in sodium chloride (NaCl) and deionised water used as negative and positive controls respectively. The percentage of RBC lysis was calculated based on the assumption that 100% of red blood cells were lysed when mixed with deionised water. The reference carboxyl functionalised CdSe/ZnS QDs were purchased from Thermo Fischer Scientific Inc. with peak red photoluminescence emission at 655 nm.

**Haemolysis test results**

In vivo haemolysis can cause pathological conditions such as anaemia and jaundice, therefore, the haemolytic potential of all intravenously administered new drugs (nanoparticles) needs to be evaluated before their clinical use (Dobrovolskaia et al. 2008b). Haemolysis activity is one of the most common tests to study nanoparticle interaction and biocompatibility with blood components. In this study, we performed haemolysis test to investigate QD-induced haemolysis of red blood cells following their incubation with bio CFQD^®^ nanoparticles at various concentrations for different incubation times. The percentage of RBC (percentage of haemolysis) was quantified relative to the positive control samples lysed with deionised water. The amount of haemoglobin released into the medium was measured spectrophotometrically. The degree of haemolysis caused by QD exposure to dilute rat blood for up to 5 hr is shown in Figure S1. The results clearly show that the bio CFQD^®^ nanoparticles did not induce haemolysis of RBC at concentrations as high as 400 nM. However, over 90 % of RBC were lysed when exposed to cadmium-based QDs.

**Figure S1**. Haemolysis of red blood cells (RBC) following incubation with QDs. RBC were incubated with bio CFQD^®^ nanoparticles and cadmium-based QDs for 5 hr at various concentrations of QDs and the haemoglobin release was measured by measuring the absorbance of the supernatant. At the 5 hr time-point the mean % haemolysis for the cadmium-based QDs is 91.5% whereas for the indium-based QDs the reading is only 5.3%. Data are mean ± SD (n=5).

**References:**

Dobrovolskaia, M.A., Clogston, J.D., Neun, B.W., Hall, J.B., Patri, A.K., & McNeil, S.E. 2008b. Method for analysis of nanoparticle hemolytic properties in vitro. *Nano.Lett.*, 8, (8) 2180-2187.
